# Development and validation of nomogram to predict severe illness requiring intensive care follow up in hospitalized COVID-19 cases

**DOI:** 10.1186/s12879-021-06656-w

**Published:** 2021-09-25

**Authors:** Rahmet Guner, Bircan Kayaaslan, Imran Hasanoglu, Adalet Aypak, Hurrem Bodur, Ihsan Ates, Esragul Akinci, Deniz Erdem, Fatma Eser, Seval Izdes, Ayse Kaya Kalem, Aliye Bastug, Aysegul Karalezli, Aziz Ahmet Surel, Muge Ayhan, Selma Karaahmetoglu, Isıl Ozkocak Turan, Emine Arguder, Burcu Ozdemir, Mehmet Nevzat Mutlu, Yesim Aybar Bilir, Elif Mukime Sarıcaoglu, Derya Gokcinar, Sibel Gunay, Bedia Dinc, Emin Gemcioglu, Ruveyda Bilmez, Omer Aydos, Dilek Asilturk, Osman Inan, Turan Buzgan

**Affiliations:** 1 Department of Infectious Disease and Clinical Microbiology, Ankara Yildirim Beyazit University, Ankara City Hospital, Bilkent Street no:1, Ankara, 06800 Turkey; 2 Department of Infectious Disease and Clinical Microbiology, Ankara City Hospital, Ankara, Turkey; 3 Department of Infectious Disease and Clinical Microbiology, University of Health Sciences, Ankara City Hospital, Ankara, Turkey; 4 Department of Internal Medicine, Ankara City Hospital, Ankara, Turkey; 5 Department of Anesthesiology and Reanimation, Ankara City Hospital, Ankara, Turkey; 6 Department of Anesthesiology and Reanimation and Intensive Care Unıt, Ankara Yildirim Beyazit University, Ankara City Hospital, Ankara, Turkey; 7 Department of Pulmonary Diseases, Ankara Yildirim Beyazit University, Ankara City Hospital, Ankara, Turkey; 8 Department of General Surgery, Ankara City Hospital, Ankara, Turkey; 9 Department of Anesthesiology and Reanimation, University of Health Sciences, Ankara City Hospital, Ankara, Turkey; 10 Department of Pulmonary Diseases, Ankara City Hospital, Ankara, Turkey; 11 Department of Microbiology, Ankara City Hospital, Ankara, Turkey

**Keywords:** COVID-19, Nomogram, Severity Score, Predictive Factors, Intensive Care

## Abstract

**Background:**

Early identification of severe COVID-19 patients who will need intensive care unit (ICU) follow-up and providing rapid, aggressive supportive care may reduce mortality and provide optimal use of medical resources. We aimed to develop and validate a nomogram to predict severe COVID-19 cases that would need ICU follow-up based on available and accessible patient values.

**Methods:**

Patients hospitalized with laboratory-confirmed COVID-19 between March 15, 2020, and June 15, 2020, were enrolled in this retrospective study with 35 variables obtained upon admission considered. Univariate and multivariable logistic regression models were constructed to select potential predictive parameters using 1000 bootstrap samples. Afterward, a nomogram was developed with 5 variables selected from multivariable analysis. The nomogram model was evaluated by Area Under the Curve (AUC) and bias-corrected Harrell's C-index with 95% confidence interval, Hosmer–Lemeshow Goodness-of-fit test, and calibration curve analysis.

**Results:**

Out of a total of 1022 patients, 686 cases without missing data were used to construct the nomogram. Of the 686, 104 needed ICU follow-up. The final model includes oxygen saturation, CRP, PCT, LDH, troponin as independent factors for the prediction of need for ICU admission. The model has good predictive power with an AUC of 0.93 (0.902–0.950) and a bias-corrected Harrell's C-index of 0.91 (0.899–0.947). Hosmer–Lemeshow test p-value was 0.826 and the model is well-calibrated (p = 0.1703).

**Conclusion:**

We developed a simple, accessible, easy-to-use nomogram with good distinctive power for severe illness requiring ICU follow-up. Clinicians can easily predict the course of COVID-19 and decide the procedure and facility of further follow-up by using clinical and laboratory values of patients available upon admission.

**Supplementary Information:**

The online version contains supplementary material available at 10.1186/s12879-021-06656-w.

## Background

The world has been under threat of the novel coronavirus disease (COVID-19) since the last days of 2019. Although the disease has a wide clinical spectrum from asymptomatic infection to critically ill [1], a small number of COVID-19 patients experience a severe illness that can result in death. The Chinese Center for Disease Control and Prevention reported mild disease, serious illness, and critical illness as 81%, 14%, and 5% in 44.672 confirmed cases, respectively [2]. The case fatality rate was reported as 2.3% but had increased to 49.0% in critical cases.

Since there is no specific treatment for the new coronavirus (severe acute respiratory syndrome coronavirus-2 [SARS-CoV-2]) so far, the early recognition of patients who will worsen and the provision of aggressive supportive treatment is the essential point of patient management. Therefore, early detection of patients whose illness will progress helps the physician to decide whether the patient should be followed up in the hospital or outpatient clinic or if there is a need for transferring to a referral center. Additionally, early detection of the disease severity with a predictive calculation tool can optimize the duration of hospitalization, especially in countries with limited resources in terms of hospital beds and finances. Early identification of patients with a simple and easy-to-use method will save time for the physicians and patients for providing rapid supportive care and reduce the mortality rate. Patients who are predicted not to need an intensive care unit (ICU) can be discharged from the hospital earlier. Herein, we aimed to construct and validate a nomogram and a web-based calculation tool that incorporated demographic, clinical characteristics, and initial laboratory results at admission to hospital for predicting the development of severe illness that will require ICU follow up.

## Methods

### Study design and participants

This retrospective cohort study was carried out in Ankara City Hospital, set apart as the main pandemic response center in Ankara with 3810 beds, of which 696 are intensive care beds. The ethical approval was obtained from Ankara City Hospital Ethical Committee 1. Verbal consent was obtained after the patients were informed that their medical records would only be used in scientific studies after anonymization of their personal information. All patients older than 18 years who were hospitalized with the diagnosis of COVID-19 infection between March 15, 2020, and June 15, 2020, were included in the study. Only COVID-19 patients with the definite diagnosis were included in the study. The diagnosis was confirmed with polymerase chain reaction (PCR) for SARS-CoV-2 performed based on the protocol established by the World Health Organization (WHO) interim guideline [3]. Patients were monitored up to June 30, 2020, the final date of follow up. Patients with a negative SARS-CoV-2 test even if typical chest computed tomography (CT) findings and those who were still in hospital at the moment of final date of follow up (if no death or discharge) were excluded.

The patients with a severe and critical illness were candidate to ICU follow-up based on the WHO COVID-19 disease severity classification. Patients with pneumonia and one of the following: > 30 breaths/min; severe respiratory distress; or O_2_ saturation (SpO_2_) < 90% on room air were considered severe. Patients were considered critical if they had acute respiratory distress syndrome (ARDS) or other respiratory failure requiring mechanical ventilation, or septic shock, and/or organ failure requiring ICU follow up [1]. The decision of ICU admission was made by intensive care specialists. ICU admission criteria were respiratory rate ≥ 30, SpO_2_ < 90% or partial oxygen pressure (PaO_2_) < 70 mmHg on room air despite nasal oxygen support of 5 lt/min or above, PaO_2_/fraction of inspired oxygen (FiO_2_) < 300. The primary outcome was defined as severe illness that required ICU follow up. The patients were classified as cases who required ICU follow up and those who did not require ICU follow up based on disease severity.

### Collecting and processing data

To collect data, a special form was created for COVID-19 patients, containing information of patients at the admission and follow up. The parameters included in special patient forms were age, gender, smoking status, comorbid diseases, the symptoms of fever and dyspnea, oxygen saturation (SpO_2_), quick sequential organ failure assessment (qSOFA) at admission. The forms also included following laboratory and radiological tests: complete blood counts, serum biochemistry, C-reactive protein (CRP), procalcitonin (PCT), coagulation tests, ferritin, D-dimer, troponin I, and chest CT. The clinical outcomes were defined as requirement of ICU or discharge from hospital.

Data were collected prospectively. In case of patient death or discharge, all the missing laboratory records in patient files were completed from the hospital database and registered in an electronic recording system and uploaded collaboratively to an online database created specifically for COVID-19 patients. Data cut-off for the study was June 30, 2020. Data were recorded to the system by the physicians who followed up the patients from different departments including infectious disease, internal medicine, respiratory disease, and anesthesiology and reanimation. After patient records were compiled, the data was checked by two independent controllers who were infectious disease physicians. Patients with more than 30% missing data were not included in the study.

### Potential predictive parameters and outcome

For the development of a functional nomogram, patient data obtained on the day of hospitalization were used. The predictors were selected from the factors that affect the prognosis of the patients such as age and the presence of comorbidities, and clinical features and easily accessible, practical, and quickly performed laboratory parameters. The potential predictive parameters were determined as age, gender, the presence of fever and dyspnea, and qSOFA on admission, clinical risk factors (comorbidities including hypertension, coronary arterial disease, diabetes mellitus, chronic lung disease, malignity, and number of comorbidities), SpO2 and laboratory parameters which are found significant covariates on COVID-19 infection including white blood cell (WBC), monocyte, neutrophil to lymphocyte ratio (NLR), hemoglobin (HGB), platelet count, urea, creatinine, glomerular filtration rate (GFR), albumin, aspartate transaminase (AST), alanine transaminase (ALT), lactate dehydrogenase (LDH), creatine kinase (CK), troponin I, CRP, PCT, ferritin, prothrombin time (PT), activated partial thromboplastin time (aPTT), D-dimer, international normalized ratio (INR) and fibrinogen, and presence of bilateral infiltration on chest CT. A total of 35 predictors were included in the construction of nomogram in the beginning. After determining potential predictors, their association with ICU hospitalization was investigated.

### Statistical analysis

Statistical analyses were performed using R software version 4.0.4 (R Foundation for Statistical Computing, Vienna, Austria) and IBM SPSS Statistics version 23.0 for Windows (IBM Corporation, Armonk, NY). Missing data pattern and mechanism were evaluated using R packages VIM, mice, MissMech, and BaylorEdPsych [4–7]. Listwise deletion (complete-case method) was applied for handling missing data due to the MNAR mechanisms in numerical measurements. Laboratory parameters were discretized using Receiver Operating Characteristic (ROC) analysis. Thereafter, Youden’s J Index was used for determining optimal cut-off points of the numerical variables in predicting ICU admission. Descriptive statistics were presented as median with interquartile range (IQR) for continuous variables since the distribution of the variables were skewed and contains extreme values. Frequency and percentages were presented as descriptive statistics for categorical variables. Mann–Whitney U test was used in the comparison of continuous variables between patients admitted to ICU and those without need for ICU follow up due to the violation of the parametric test assumptions. Pearson χ2 test was used for testing independence between ICU admission status and other categorical variables when test requirements were satisfied. Otherwise, Fisher’s Exact test was used.

To estimate ICU admission status, univariate logistic models were constructed using bootstrap sampling with 1000 samples and bootstrap estimated p values were evaluated. The variables with bootstrapped p-value below 0.25 as considered candidate variable for multivariable analysis [8]. Numeric variables which were included in the multivariable analysis are evaluated for linearity in logit and multicollinearity was investigated using Variance inflation factor (VIF) before applying the variable selection method [9]. Both univariate and multivariable logistic regression analysis were carried out using R rms package [10]. Fast backward elimination method for variable selection was carried out with bootstrap sampling (1000 successful bootstrap samples) to develop a parsimonious model for predicting ICU admission. Estimations obtained from multivariable model was based on penalized maximum likelihood estimations with best penalty parameter obtained using pentrace function in R rms package. In addition, final model is selected according to the Akaike Information Criteria (AIC).

Selected variables were represented as odds ratio (OR) with bootstrapped 95% confidence interval (CI) and two-tailed *p*-values. Discrimination was evaluated using bias-corrected Harrell’s Concordance index (C-index). Bias-corrected Harrel’s C-index was calculated from rms package validate function with 1000 successful bootstrap samples. Validated model is checked for multicollinearity. Hence, VIF values of all the predictor variables in the multivariable model were below 5. In addition, linearity in logit assumption was satisfied. Nomogram was constructed based on the final validated model for estimating the admission to ICU and provided a quantitative tool for physicians to assess the individual probability of ICU admission. In addition, model’s discriminative power was evaluated with ROC analysis using R pROC package [11]. The Area Under the Curve (AUC) was obtain with 95% Hereafter in the article, ‘corrected C-index” will be used to indicate that AUC value/C-index was obtained from bootstrap samples and bias-corrected, and “AUC” will be used to indicate crude AUC value/C-index which was obtained from one ROC Curve.

Calibration plots were developed to assess the predictive accuracy and agreement between predicted and observed ICU admission with 1000 bootstrap samples and calibration curve analyses were performed in addition to Hosmer–Lemeshow goodness of fit evaluation. In addition, both the unreliability test and the calibration test are performed to evaluate good calibration.

In addition, false-negative (i.e. not admitting the patient in the ICU when the patient needs intensive care) is far more harmful than the false positive (i.e. admitting the patient in the ICU when the patient doesn’t need intensive care at all) in the present study. Therefore, decision curve analysis was performed since the possibility of ICU need of the patient is more crucial for patients' well-being. In a decision curve analysis, a low-risk threshold probability might indicate that delaying the ICU admission is far more harmful than early admission; a higher threshold might indicate that waiting the parameters to reach critical levels is relatively more harmful than unnecessary ICU admission.

All analysis related to the evaluating classification performance and calibration of the prognostic accuracy of the nomogram model were performed according to TRIPOD guidelines [12].

## Results

### Characteristics of study cohort

**Fig. 1:**
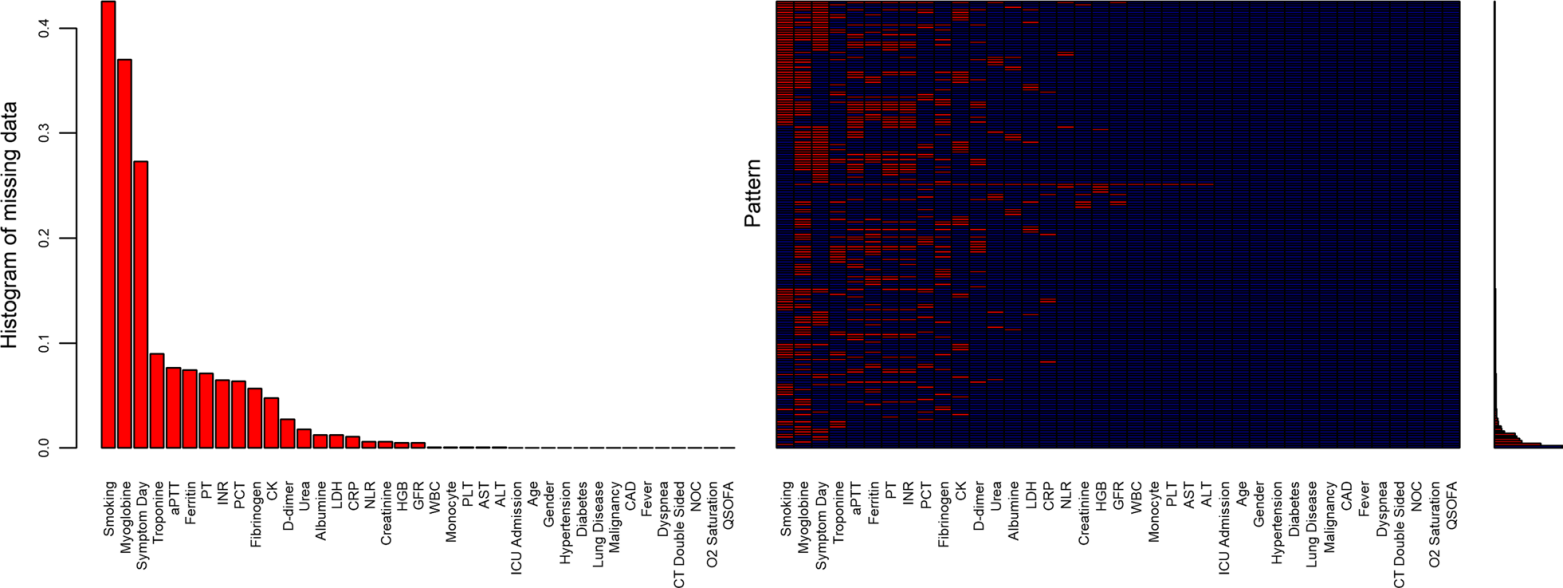
Evaluation of missing data mechanism. The first plot represents the distribution of the missing data proportions in the variables considered in the study. The second plot is a heatmap represent the missing data pattern in the study. The third plot is the vertical version of the first plot which represents the missing data proportions in the heatmap

Study included 1022 patients with laboratory confirmed COVID-19. The dataset consists of these 1022 patient records contained missing data ranged between 0.1% and 42%. Variables which consist of more than 20% percent of missing data were excluded from the analysis. The proportion of missing data ranged from 0.1% to 9% after exclusion of the variables which consist of more than %20 missing observations (smoking status, myoglobin, symptom duration before hospital admission). The MCAR test in the R MissMech package [4–7] was used to assess whether the missing data mechanism is Missing Completely at Random (MCAR). MCAR hypothesis was rejected at 0.05 level. Therefore, after list-wise deletion 686 cases out of 1022 patient remained for further analysis. Brief overview of the missing data structure is represented graphically (Fig. 1).

The demographic characteristics, comorbidities, and initial laboratory parameters of patients are shown in Table 1. Of the 686 patients, 104 (15.2%) required ICU follow up during hospitalization. There was no difference in gender between the groups (p = 0.057), 52.4% of patients who did not need ICU follow up and 62.5% of those who needed ICU follow up were male. The median age was higher in the patients requiring ICU follow up (67, IQR 30–54) than those who did not need ICU follow up (42, IQR 54–76) (p < 0.001). The patients who required ICU follow up had significantly higher rates of hypertension (40.4% vs 12.8%), coronary arterial disease (20.2% vs 6.4%), diabetes mellitus (29.3% vs 10.1%), chronic pulmonary disease (21.2% vs 6.4%), and malignity (9.6% vs 1.9%) compared to those who did not need ICU admission (for all, p < 0.001). Fever (48.1% vs 34.0%, p = 0.006) and dyspnea (59.9% vs 19.4%, p < 0.001) were significantly more frequent on admission in the patients who needed ICU follow up. Laboratory features of two groups were compared. All initial parameters except ALT and aPTT were significantly different between the two groups (for all parameters, p < 0.001).

**Table 1:** Demographic, clinical characteristics and laboratory parameters of COVID-19 patients

	Patient did not need ICU follow up (N = 582)	Patient need ICU follow up (N = 104)	p
Age. median (IQR), years	42 (30–54)	67 (54–76)	< 0.001
Age, years			< 0.001
≥ 56.5	121 (20.8)	76 (73.1)	
< 56.5	461 (79.2)	28 (26.9)	
Gender, female sex	277 (47.6)	39 (37.5)	0.057
Hypertension	106 (12.8)	42 (40.4)	< 0.001
Diabetes	58 (10.1)	31 (29.3)	< 0.001
Chronic pulmonary disease	37 (6.4)	22 (21.2)	< 0.001
Malignity	11 (1.9)	10 (9.6)	< 0.001
Coronary arterial disease	37 (6.4)	21 (20.2)	< 0.001
Fever on admission	198 (34.0)	50 (48.1)	0.006
Dyspnea on admission	113 (19.4)	62 (59.9)	
Oxygen saturation, %			< 0.001
> 94.5	463 (79.5)	22 (21.2)	
≤ 94.5	119 (20.4)	82 (78.8)	
qSOFA			< 0.001
0	553 (95.0)	38 (36.5)	
≥ 1	29 (5.0)	66 (63.5)	
White blood cell, × 10^9^/L			0.019
≥ 7.4	85 (14.6)	36 (34.6)	
< 7.4	497 (85.4)	68 (65.5)	
Monocyte, × 10^9^/L			0.006
> 0.23	506 (86.9)	70 (67.5)	
≤ 0.23	76 (13.1)	34 (32.7)	
NLR			< 0.001
≥ 3.5	173 (29.7)	77 (74.0)	
< 3.5	409 (70.3)	27 (26.0)	
Hemoglobin, g/L			< 0.001
> 12.5	458 (78.7)	61 (58.7)	
≤ 12.5 g/L	124 (21.3)	43 (41.3)	
Platelet count, × 10^9^/L			0.031
> 155	492 (84.5)	73 (70.2)	
≤ 155	90 (15.5)	31 (29.9)	
GFR, ml/min/1.73 m2			< 0.001
> 96.2	369 (63.4)	24 (23.1)	
≤ 96.2	213 (36.6)	80 (76.9)	
Aspartate transaminase, U/L			< 0.001
≥ 28.5	166 (22.5)	79 (76.0)	
< 28.5	416 (71.5)	25 (24.0)	
Alanine transaminase, U/L			0.164
≥ 40.5	128 (22.0)	33 (317)	
< 40.5	454 (78.0)	71 (68.3)	
Albumin, g/L			< 0.001
> 42.5	417 (71.5)	23 (22.1)	
≤ 42.5	166 (28.5)	81 (77.9)	
Creatine kinase, U/L			0.002
≥ 141.5	136 (23.4)	45 (43.3)	
< 141.5	446 (76.6)	59 (56.7)	
Lactate dehydrogenase, U/L			< 0.001
≥ 286.5	94 (16.2)	71 (68.3)	
< 286.5	488 (83.8)	33 (31.7)	
C-reactive protein, g/L			< 0.001
≥ 0.0275	123 (21.1)	81 (77.9)	
< 0.0275	459 (78.9)	23 (22.1)	
Procalcitonin, pg/mL			< 0.001
≥ 0.085	71 (12.2)	71 (68.7)	
< 0.085	511 (87.8)	33 (31.7)	
Troponin, ng/L			< 0.001
≥ 5.9	113 (19.4)	78 (75.0)	
< 5.9	469 (80.6)	26 (25.0)	
Prothrombin time, sec			< 0.001
≥ 12.5	153 (26.3)	63 (60.6)	
< 12.5	429 (73.7)	41 (39.4)	
aPTT, sec			0.499
≥ 26.2	162 (27.8)	40 (38.5)	
< 26.2	72.8 (72.2)	64 (61.5)	
INR			< 0.001
≥ 1.06	183 (31.4)	68 (65.4)	
< 1.06	399 (68.6)	36 (34.6)	
Ferritin, µg/L			< 0.001
≥ 227.5	155 (26.6)	70 (673)	
< 227.5	427 (73.4)	34 (32.7)	
D-dimer, mg/L			< 0.001
≥ 0.535	210 (36.1)	88 (84.6)	
< 0.535	372 (63.9)	16 (15.4)	
Fibrinogen, g/L			< 0.001
≥ 3.40	210 (36.1)	76 (73.1)	
< 3.40	372 (63.9)	28 (26.9)	
Urea, mg/dL			< 0.001
≥ 36.1	116 (19.9)	56 (53.8)	
< 36.1	466 (80.1)	48 (46.2)	
Creatinine, mg/dL			< 0.001
≥ 0.96	125 (21.5)	41 (39.4)	
< 0.96	457 (78.5)	63 (60.6)	
Bilateral infiltration on CT	250 (43.0)	79 (70.6)	< 0.001

IQR, Interquartile range; NLR, Neutrophil to lymphocyte ratio; aPTT, Activated partial thromboplastin time; INR, International normalized ratio; CT, Computed tomography; GFR, Glomerular filtration rate; qSOFA, quick sequential organ failure assessment

Univariate analysis indicates that common laboratory features have possible effect on the patient’s requirement of intensive care as well as the patient characteristics such as age, gender and comorbidities (Table 2).

**Table 2:** Univariate analysis of potential predictive parameters for ICU admission in hospitalized COVID-19 patients

	Beta	OR	95% CI L	95% CI U	p
Age, 56.5 years and older	2.34	10.34	6.42	17.78	< 0.0001
Gender. female sex	−0.42	0.66	0.43	1.03	0.0577
Hypertension	1.11	3.04	1.96	4.91	< 0.0001
Diabetes	1.35	3.84	2.30	6.27	< 0.0001
Chronic pulmonary disease	1.37	3.95	2.01	6.98	< 0.0001
Malignity	1.71	5.52	2.04	13.65	0.0004
Coronary arterial disease	1.32	3.73	2.00	6.42	< 0.0001
Number of comorbidities	0.81	2.24	1.63	3.05	< 0.0001
Fever on admission	0.59	1.80	1.14	2.75	0.0098
Dyspnea on admission	1.81	6.13	3.87	9.65	< 0.0001
Oxygen saturation, 94.5% and below	2.67	14.50	8.93	25.80	< 0.0001
White blood cell, 7.4 × 10^9^/L and above	1.13	3.10	1.83	5.00	< 0.0001
Monocyte, 0.23 × 10^9^/L and below	1.17	3.23	1.95	5.28	< 0.0001
NLR, 3.5 and above	1.91	6.74	4.25	11.37	< 0.0001
Hemoglobin, 12.5 g/L and below	0.96	2.60	1.69	4.02	< 0.0001
Platelet count, 155 × 10^9^/L and below	0.84	2.32	1.41	3.67	0.0006
Urea, 36.1 mg/dL and above	1.55	4.69	3.06	7.42	< 0.0001
Creatinine, 0.96 mg/dL and above	0.87	2.38	1.54	3.69	< 0.0001
GFR, 96.2 ml/min/1.73 m2 and below	1.75	5.78	3.47	9.68	< 0.0001
Aspartate transaminase, 28.5 U/L and above	2.07	7.92	5.02	13.39	< 0.0001
Alanine transaminase, 40.5 U/L and above	0.50	1.65	1.02	2.64	0.0369
Albumin, 42.5 g/L and below	2.18	8.83	5.66	15.52	< 0.0001
Creatine kinase, 141.5 U/L and above	0.92	2.50	1.61	3.85	< 0.0001
Lactate dehydrogenase, 286.5 U/L and above	2.41	11.17	6.94	18.72	< 0.0001
C-reactive protein, 0.0275 g/L and above	2.58	13.14	8.42	22.96	< 0.0001
Procalcitonin, 0.085 pg/mL and above	2.74	15.49	10.19	25.67	< 0.0001
Troponin, 5.9 ng/L and above	2.52	12.45	7.90	22.09	< 0.0001
Prothrombin time, 12.5 s and above	1.46	4.31	2.86	6.52	< 0.0001
aPTT, 26.2 s and above	0.48	1.62	1.05	2.43	0.0230
INR, 1.06 and above	1.42	4.12	2.67	6.54	< 0.0001
Ferritin, 227.5 µg/L and above	1.74	5.67	3.73	9.13	< 0.0001
D-dimer, 0.535 mg/L and above	2.28	9.74	5.95	18.92	< 0.0001
Fibrinogen, 3.40 g/L and above	1.57	4.81	3.10	7.94	< 0.0001
Bilateral infiltration on CT	1.43	4.20	2.68	7.33	< 0.0001
qSOFA:1	2.67	14.49	9.38	22.95	< 0.0001
qSOFA:2	3.26	25.92	15.96	36.14	< 0.0001

NLR, Neutrophil to lymphocyte ratio; aPTT, Activated partial thromboplastin time; INR, International normalized ratio; CT, Computed tomography; GFR, Glomerular filtration rate; qSOFA, quick sequential organ failure assessment

### Potential predictive factors for ICU admission

A total of 35 predictor were chosen for the development of nomogram predicting of ICU admission in hospitalized patients with COVID-19. All predictors have p-value below 0.25. Therefore, they were all considered as candidate variables for multivariable analysis except QSOFA score due to sparsity and quasi-complete separation problem (All the patients whose QSOFA score equals 2 had been admitted to the ICU).

### Construction of nomogram predicting ICU admission status

**Fig. 2:**
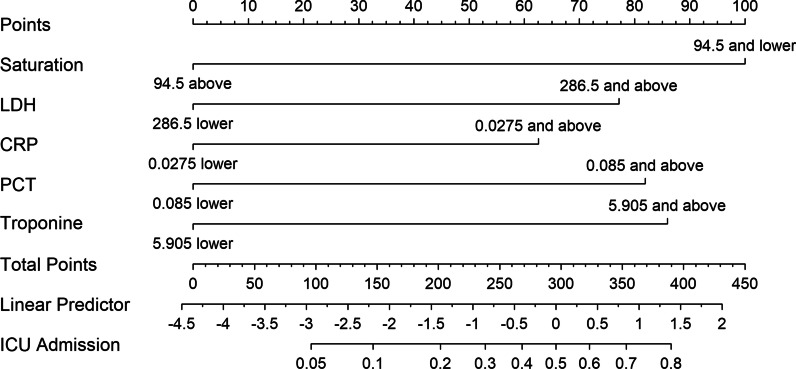
Nomogram predicting the risk of ICU need in hospitalized COVID-19 patients based on patients’ characteristics on the first admission. The nomogram included oxygen saturation, CRP, PCT, LDH and troponin. The total point is obtained with the sum of assigned points per each parameter. CRP:C-reactive protein, PCT: procalcitonin, LDH: lactate dehydrogenase, ICU: intensive care unit

The nomogram was constructed using the data obtained from 686 patients’ records. Afterwards, all laboratory parameters were discretized by using optimal cut-off points obtained from ROC analysis with Youden’s J Index. To construct multivariable nomogram model for estimating ICU admission status of inpatients, first candidate variables were selected using univariate analysis (Table 2). All variables had p value below 0.25 and considered for the multivariable analysis. After candidate variables were chosen, multivariable model was constructed with 34 variables including demographical, clinical and laboratory parameters. The parameters included in the initial model were age, gender, hypertension, coronary arterial disease, diabetes mellitus, chronic pulmonary disease, malignity, number of comorbidities, fever and dyspnea on admission, SpO2, WBC, monocyte, NLR, HGB, platelet count, urea, creatinine, GFR, AST, ALT, albumin, LDH, CK, CRP, PCT, ferritin, troponin, PT, aPTT, INR, D-dimer, fibrinogen and bilateral infiltration on CT. Final model was selected according to the Akaike Information Criteria (AIC), and only SpO2, LDH, CRP, PCT and troponin, which were shown to be independent risk factors for predicting ICU admission, were included. The present nomogram calculates the risk for requirement of ICU in hospital admission of patients using these 5 parameters (Fig. 2). Additional information on score assignment for each variable and calculation the risk for ICU admission based on total point is shown in Additional file 1. According to the nomogram model, the risk of ICU admission is 4.4 (95% CI 2.48–7.72) times higher in patients with oxygen saturation equal and below 94.5% compared to the patients with oxygen saturation above 94.5% (p < 0.0001). In addition, the risk is 3.1 (95% CI 1.76–5.53) times higher in patients with LDH level equal and above 286.5 U/L compared to the patients with LDH level below 286.5 U/L (p < 0.0001) while it is 2.5 (95% CI 1.37–4.63) times higher in patients with CRP level equal and above 0.0275 g/L compared to the patients with CRP level below 0.0275 g/L (p = 0.0029). Whereas the risk is 3.4 (95% CI 1.89–5.94) times higher in patients with PCT level equal and above 0.085 pg/mL compared to the patients with PCT level below 0.085 pg/mL (p < 0.0001), it is 3.6 (95% CI 2.03–6.22) times higher in patients with troponin level equal and above 5.9 ng/L compared to the patients with troponin level below 5.9 ng/L (p < 0.0001) (Table 3).

**Table 3:** Estimation of the Odds ratios with 95% confidence intervals of the multivariable nomogram model

Variable	Odds ratio	%95 CI lower bound	%95 CI upper bound	p
Intercept	0.013	0.0069	0.0236	< 0.0001
Oxygen saturation (SpO2), 94.5% and below	4.372	2.4769	7.7168	< 0.0001
Lactate dehydrogenase (LDH), 286.5 U/L and above	3.122	1.7636	5.5248	< 0.0001
C-reactive protein (CRP), 0.0275 g/L and above	2.518	1.3706	4.6274	0.0029
Procalcitonin (PCT), 0.085 pg/mL and above	3.347	1.8878	5.9347	< 0.0001
Troponin, 5.9 ng/L and above	3.555	2.0318	6.2195	< 0.0001

CI, Confidence Interval

Based on these 5 independent risk factors included in the nomogram for ICU admission, a web-based calculation tool was constructed. The clinician can easily access the calculation tool using the online website at https://achcovid19.com/prj/f?p=126:1.

### The accuracy of nomogram prediction model validation

**Fig. 3:**
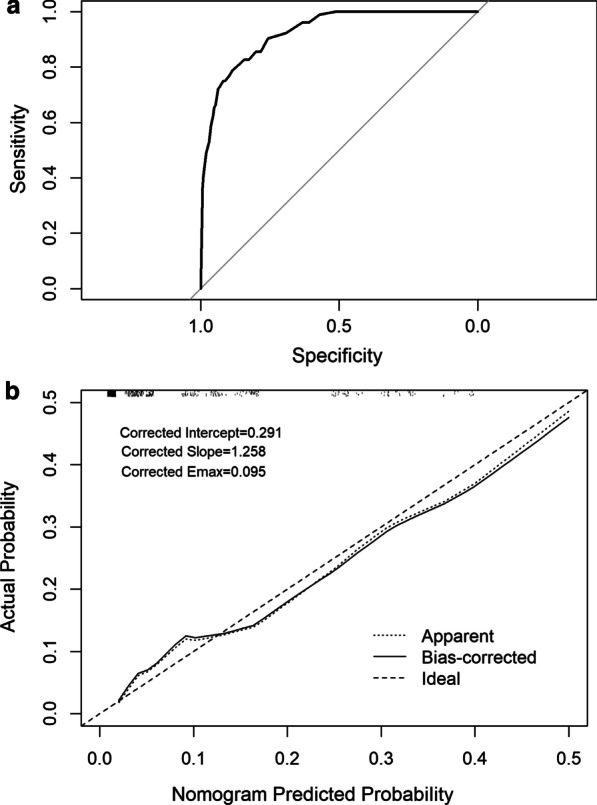
Receiver-operating characteristics (ROC) (**a**) and calibration curves (**b**) analysis of the nomogram

The nomogram model had a significantly high predictive value for the development of ICU needs in hospitalized patients. The model had an AUC of 0.93 (0.902–0.950) (Fig. 3a). In addition, we evaluate the validation of the final model using bootstrap resampling method and obtained corrected C-index of the nomogram as 0.91 (95% CI 0.899–0.947) which implies exceptionally good discriminative value for differentiating inpatients who needed ICU follow up from those who did not.

**Fig. 4:**
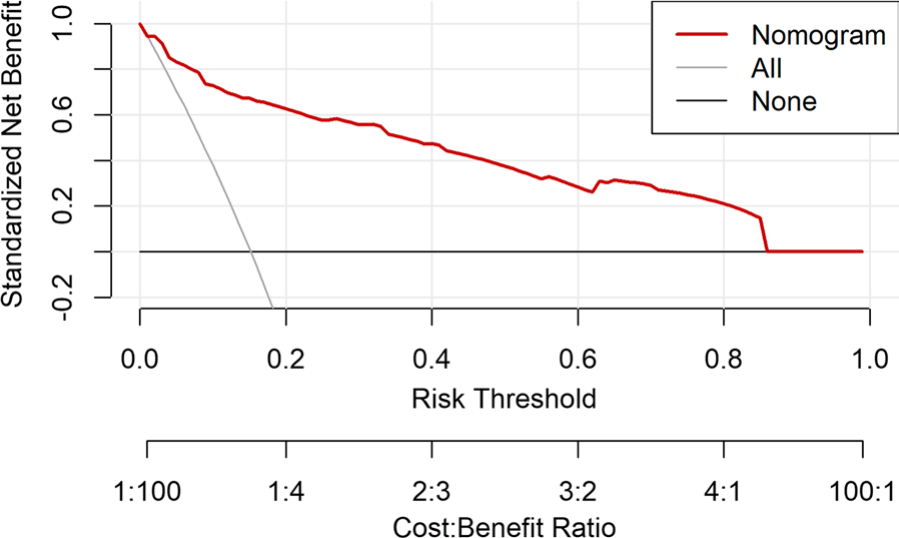
Decision curve analysis of the nomogram predicting the risk of ICU need in hospitalized COVID-19 patients. The x-axis indicates the threshold probability, y-axis measures net benefit by adding true positive and subtracting false positive. A low-risk threshold probability might indicate that delaying the ICU admission is far more harmful than early admission; a higher threshold might indicate that waiting the parameters to reach critical levels is relatively more harmful than unnecessary ICU admission. When, threshold probability between 0.15–0.85, predicting ICU admission by using our nomogram model would provide higher benefit than the admitting all the patients to the ICU (All i.e. treat all) or admitting none of the patients to the ICU (None i.e. treat none)

Furthermore, the decision curve revealed that when threshold probability is between 0.15 and 0.85, predicting ICU admission by using our nomogram model would provide higher net benefit than the admitting all the patients to the ICU (All) or admitting none of the patients to the ICU (None) (Fig. 4).

### Calibration

Hosmer–Lemeshow goodness of fit test indicated that there is no significant difference between the predictive calibration curve and the ideal curve for predicting the ICU status of the patients (X-squared = 4.3284, df = 8, p-value = 0.8263) (Fig. 3b). The calibration curves and Hosmer–Lemeshow test results indicates that the nomogram model is calibrated. In addition, significance of miscalibration of the model is evaluated using unreliability test (p = 0.8197) and calibration test (p = 0.1703) which indicates that the model is statistically well-calibrated.

## Discussion

As it is known, COVID-19 has a mild-to-moderate course in most patients, but it progresses to severe illness in one-fifth of patients. Knowing which patients COVID-19 will have a severe course is crucial in the management of patients and optimal use of medical resources such as hospital beds. Early identification of COVID-19 patients at high risk for serious disease development will enable patients to reach faster supportive care and treatment. On the other hand, determining patients with low risk of developing ICU need can assist physicians in the decision of discharge of these patients. Especially, the healthcare facilities with a low capacity of ICU beds have difficulties in the follow-up of patients. These facilities can prefer to transfer the patients with high risk for the development of ICU need to the further healthcare facilities with high capacity of ICU beds, while they can follow-up the patients with low risk for ICU need in their hospital wards. Therefore, we developed a simple and easy-to-use nomogram (and online calculator) that makes physician’s decisions on the management of COVID-19 patients easier and provides ways of recognizing severe illness requiring ICU by using available and accessible values of patients on the first admission.

Several risk factors associated with the severity of disease have so far been reported in COVID-19 patients [13–17]. However, evaluating these risk factors by using a nomogram that predicts the severity of the disease can be more realistic and practical method for physicians. Nomograms are simple calculators, used commonly in the medicine, that could predict an individual numerical probability of a clinical event [9]. We constructed and validated a functional nomogram that incorporated five variables to predict the patients that carry a high risk for ICU admission by using baseline demographic, clinical, and laboratory parameters of the patients. The strongest nomogram was obtained with five independent variables consisting of SpO2, CRP, PCT, LDH, and troponin. The present nomogram has an excellent discriminative value with an AUC of 0.93 in the prediction of the individual risk of ICU admission in hospitalized patients with COVID-19. We demonstrated the optimal performance of the model by validation. As these five components are easily accessible parameters in the emergency department, this nomogram can help physicians to classify patients properly and decide on the proper follow up strategy.

In our study, the parameter with the greatest impact on ICU admission is SpO2 (100 points). As it is known, COVID-19 is a respiratory tract disease that can cause multisystem involvement and hypoxia is one of its most expected symptoms that also indicates the severity of the disease [1]. We demonstrated that the need of ICU admission is 4.4 (95% CI 2.48–7.72) times higher in patients with a SpO2 of 94.5% and below compared to others. Saturation was reported as an independent risk factor for mortality in COVID-19 patients [18, 19]. Like our study, Acar et al. reported a 2.81-fold increase in mortality in patients having SpO2 between 89 and 94% and an 8.81-fold increase in SpO2 of 88% or less. Dyspnea and tachypnea, which are indicators of hypoxia, have also been reported to be associated with disease severity and unfavorable outcomes [20–22]. Hyperinflammation and its impact on severe COVID-19 was shown in COVID-19 pandemic [23]. Both CRP and PCT are inflammatory parameters and reported to be associated with severe illness or mortality in patients with COVID-19 in many studies [16, 19, 21, 24–30]. In this study, CRP and PCT were detected as independent factors associated with increased ICU admission risk and both were included in the nomogram. Although not identical, our results contain similar findings with previous reports. In our study, the risk for ICU need is 2.5 (95% CI 1.37–4.63) times higher in patients with a CRP of 0.0265 g/L or more when compared to those having lower CRP. Previous studies detected different cut-off values and ranges for CRP as a predictor of disease severity [19, 21, 24–27].

In our model, LDH is another marker to predict ICU need. The patients with an LDH of 286.5 U/L and above had 3.1 (95% CI 1.76–5.53) times higher risk for severe infection requiring ICU follow up. LDH is a tissue damage marker and is released from cells into the serum in the existence of cell damage. Therefore, LDH may help detecting tissue damage in the onset of COVID-19 infection. Similar to our study, some previous studies reported LDH as a predictive marker for severe illness [20, 24, 27, 31, 32]. Troponin I is also one of the predictors in our nomogram. There is growing evidence on the unfavorable impact of cardiac events associated with COVID-19 on prognosis. Some studies reported that troponin I was detected significantly higher in those who died or needed mechanical ventilation compared to survivors or patients who did not need mechanical ventilation [33, 34]. Troponin I may be an early indication of worsening in COVID-19 patients without a detectable cardiac event.

In the literature, there are studies proposing nomograms and/or different models to predict serious illness or death in COVID-19 patients [19, 21, 22, 24, 25, 35–37]. Age was reported as a predictor for severe COVID-19 or mortality in previous studies and is included in most of the prediction models [16–21, 24, 25, 32, 35, 38, 39]. We found an increased risk of severe infection requiring ICU admission in the patients 56.5 years of age and older. However, it was not found significant enough to be included in the final nomogram as an optimal predictor for severe illness requiring ICU follow up. The nomogram developed by Gong et al. for early identification of cases with a high risk of progression to severe COVID-19 included older age with six laboratory parameters [24]. Yu et al. developed a nomogram incorporated age and chest CT characteristics to define severe COVID-19 in non-severe hospitalized COVID-19. Liang W et al. reported that they developed and validated a clinical risk score named as COVID-GRAM with ten parameters (chest radiographic abnormality, hemoptysis, dyspnea, age, unconsciousness, number of comorbidities, cancer histories, neutrophil-to-lymphocyte ratio, LDH, and direct bilirubin) [20]. However, like our study, there are also studies in which age was not determined as a predictor for disease severity [22, 26, 28, 40]

In addition to older age, the presence of comorbidities such as hypertension, cardiovascular disease and diabetes are risk factors for a severe disease requiring ICU in COVID-19 patients [16, 17, 31]. The association between comorbid diseases and the development of severe infection was indicated in previous studies [14, 16, 17, 31, 41, 42]. Some were severity and mortality risk scores [16, 19, 22, 31, 32, 37]. Although we found that comorbid diseases were more frequent in severe cases requiring ICU follow up than the patients without ICU need, comorbidities were not identified as optimal predictors during the development process of the nomogram.

There are many strengths of our study. It is demonstrated that COVID-19 is a multisystem disease with uncontrolled inflammatory response and tissue damage. Therefore, finding different parameters from different pathways; SpO2 representing the respiratory system, CRP and PCT as inflammatory markers, LDH as markers of tissue damage, and troponin from cardiac involvement, proves the good fitness of our model to this disease’s pathogenesis. Multi-disciplinary nature of our model provides the opportunity to make an integrated decision related with the follow-up strategy for the patient. These five parameters can easily be obtained in emergency departments or outpatient clinics. The nomogram in our study exhibited an good discriminative power with an corrected C-index of 0.91 in the prediction of severe illness requiring ICU follow up on admission. Its performance is calibrated [10, 43, 44].

Our study has some limitations. Firstly, some patients are directly admitted to the ICU or immediately transferred to ICU on admission. To overcome this problem, we did not include patients who were directly hospitalized in the intensive care unit or those who were transferred to intensive care within the first 24 h of hospitalization. Secondly, we performed the study in a referral center. The more extensive, multicenter, and large sample sized studies will be better to represent the whole population. Thirdly, the study was designed retrospectively. Some cases had incomplete data. A large number of patients could not be included in the model development process and others had also missing data in acceptable limits.

## Conclusion

We developed a nomogram for the prediction of severe illness requiring ICU with good distinctive power. The present nomogram supports the clinician through available clinical and laboratory parameters obtained at the first admission. The clinician can decide more easily where the patient should be followed, in the hospital or outside (at home, in the isolation institute or nursing home), or further healthcare facilities. The primary or secondary care facilities can use the present nomogram when they first examine the patients to decide whether the patients have a high risk for the development of ICU need or not, or whether they should transfer the patients or not. The patients who have severe illness or have a potential for worsening in the following days can be transferred earlier to the appropriate clinic. The patients with low risk for severe illness and ICU need can be more confidently discharged from hospital in facilities with low-bed capacity due to not expecting that patient’s disease will not probably progress to severe illness. Finally, the optimal use of hospital beds can be provided by preventing unnecessarily long hospitalizations in those who were predicted low risk for the severe outcome, especially in countries with limited sources in terms of hospital beds or financial capacity.

## Supplementary Information

**Additional file 1: Table S1.** Score assignment for each variable included in nomogram and calculation the risk for ICU admission based on total point. **Table S2.** Calibration indexes of the nomogram model.

## Data Availability

The anonymized datasets generated and/or analyzed during the current study are not publicly available due to Turkey’s Personal Data Protection Law No. 6698, but are available from the corresponding author upon reasonable request.

## Abbreviations

COVID-19: Coronavirus Disease-2019
SARS-CoV-2: Severe Acute Respiratory Syndrome Coronavirus-2
CT: Computer tomography
WHO: World Health Organization
ARDS: Acute respiratory distress syndrome
PCR: Polymerase chain reaction assay
ICU: Intensive care unit
SD: Standard deviation
IQR: Inter-quartile range
OR: Odds ratio
CI: Confidence interval
