# Supplementary material for: Development and validation of nomogram to predict severe illness requiring intensive care follow up in hospitalized COVID-19 cases
Source: BMC Infect Dis. 2021 Sep 25;21:1004. doi: 10.1186/s12879-021-06656-w (PMC8467006; doi:10.1186/s12879-021-06656-w)
Supplement: Supplementary file 1 — Additional file 1: Table S1. Score assignment for each variable included in nomogram and calculation the risk for ICU admission based on total point. Table S2. Calibration indexes of the nomogram model. [file 12879_2021_6656_MOESM1_ESM.docx]

**Table S1. Score assignment for each variable included in nomogram and calculation the risk for ICU admission based on total point**

| **Variables** | | | **Point Per Variable** |
| --- | --- | --- | --- |
| Saturation, % | |  | |
| ≤ 94.5 | | 0 | |
| > 94.5 | | 100 | |
| CRP, g/L | |  | |
| < 0.0275 | | 0 | |
| ≥ 0.0275 | | 63 | |
| PCT, pg/mL | |  | |
| < 0.085 | | 0 | |
| ≥ 0.085 | | 82 | |
| LDH, U/L | |  | |
| < 286.5 | | 0 | |
| ≥ 286.5 | | 77 | |
| Troponin I, ng/L | |  | |
| < 5.9 | | 0 | |
| ≥ 5.9 | | 86 | |
| **Total point** | | | **Risk for ICU admission** |
| 96 | 0.05 | | |
| 147 | 0.10 | | |
| 202 | 0.20 | | |
| 238 | 0.30 | | |
| 268 | 0.40 | | |
| 296 | 0.50 | | |
| 323 | 0.60 | | |
| 353 | 0.70 | | |
| 390 | 0.80 | | |

**Table S2. Calibration indexes of the nomogram model**

|  | **Index** | **Corrected Index** |
| --- | --- | --- |
| **D_xy_** | 0.8513 | 0.8146 |
| **R^2^** | 0.4819 | 0.5202 |
| **Intercept** | 0.0000 | 0.2908 |
| **Slope** | 1.0000 | 1.2583 |
| **E_max_** | 0.0000 | 0.0955 |
| **D** | 0.3217 | 0.3531 |
| **U** | -0.0029 | 0.0084 |
| **Q** | 0.3246 | 0.3447 |
| **B** | 0.0704 | 0.0774 |
| **g** | 1.5605 | 1.9875 |
| **gp** | 0.1877 | 0.2106 |

*Indexes obtained from 1000 bootstrap samples. D_xy_: Somers’ D_xy_ rank correlation; R^2^: R-squared index; Intercept and Slope: the intercept and slope of an overall logistic calibration equation; E_max_: the maximum absolute difference in predicted and calibrated probabilities; D: the discrimination index; U: Unreliability index; Q: the overall quality index; B: the Brier score; g: g-index; gp: g-index on the probability scale [10]
